# Genome Sequence of Linnemannia hyalina Strain SCG-10, a Cold-Adapted and Nitrate-Reducing Fungus Isolated from Cornfield Soil in Minnesota, USA

**DOI:** 10.1128/MRA.00692-21

**Published:** 2021-09-16

**Authors:** Nouf Aldossari, Satoshi Ishii

**Affiliations:** a Department of Soil, Water, and Climate, University of Minnesota, Saint Paul, Minnesota, USA; b BioTechnology Institute, University of Minnesota, Saint Paul, Minnesota, USA; Vanderbilt University

## Abstract

We report here the genome sequence of Linnemannia hyalina strain SCG-10, a cold-adapted and nitrate-reducing fungus isolated from soil. The genome of strain SCG-10 (51.6 Mbp) contained 12,693 protein-coding sequences.

## ANNOUNCEMENT

Some fungi can reduce nitrate or nitrite to gaseous forms of nitrogen via fungal denitrification (1). The fungal nitrite reductase gene (*nirK*) and the cytochrome P450 nitrite reductase gene (*p450nor*) are considered the key genes for fungal denitrification (1). While 12% and 23% out of >700 fungal genomes contain *nirK* and *p450nor*, respectively (2), only a few of these fungi have been experimentally verified as being denitrifiers.

We previously isolated 91 nitrate-reducing fungal strains from woodchip bioreactors and the adjacent cornfield soil in Minnesota, USA (3). One of the strains, Linnemannia hyalina strain SCG-10, can reduce ^15^N-labeled nitrate to ^30^N_2_ at cold temperature (5°C) and therefore has strong potential for bioaugmentation applications. However, *nirK* and *p450nor* are not detected by PCR (3). To detect these genes and other genes important for denitrification, we sequenced the whole genome of Linnemannia hyalina strain SCG-10.

Genomic DNA was extracted from a 3-g pellet of cells grown in glycerol peptone broth supplemented with 2 g/liter of sodium nitrate (3) at 5°C for 1 week. The cells were frozen in liquid nitrogen and homogenized using a micropestle before DNA extraction using the DNeasy PowerSoil kit (Qiagen, Hilden, Germany). Genomic DNA was sent to GENEWIZ (South Plainfield, NJ, USA) for genome sequencing. The SMRTbell libraries were prepared using the SMRTbell Express template prep kit v1.0 (PacBio, Menlo Park, CA, USA) per the manufacturer’s protocol. The pooled library was bound to polymerase using the Sequel binding kit v3.0 (PacBio) and loaded onto a PacBio Sequel instrument using the Sequel sequencing kit v3.0. Sequencing was performed on the required PacBio Sequel single-molecule real-time (SMRT) cells. Sequel DNA Internal Control v3.0 (PacBio) was used for quality control purposes. A total of 623,266 reads (>13.7 Gbp) were produced with a mean polymerase read length of 21,933 bp. After removing adapter sequences, the reads were assembled using HGAP4 with a genome length setting of 50 Mb and annotated using Funannotate v1.8.1 (https://funannotate.readthedocs.io/en/latest/) (4) to 52 contigs with an *N*_50_ value of 2,317,658 bp. Default parameters were used except where otherwise noted.

The total genome size was identified as 51,558,230 bp with a GC content of 48.28%. The genome contains 12,693 predicted protein-coding sequences and 317 tRNAs. We tried to find the homologs for fungal NirK and P450 Nor in the genome of strain SCG-10 by running BLASTp v2.8.1 and using the NirK and P450 Nor of Fusarium oxysporum as the queries (GenBank accession no. ABU88100 and BAA03390, respectively). However, these proteins were not identified in the genome of strain SCG-10 based on the E value cutoff of 10^−5^, indicating that the nitrate reduction and N_2_ production capability of strain SCG-10 may not be directly related to denitrification or that previously unknown genes may be involved in the process. Further experiments (e.g., transcriptome sequencing [RNA-seq]) might be helpful to identify the genes important for nitrate reduction and N_2_ production.

### Data availability.

The complete genome sequence of Linnemannia hyalina is available in the DDBJ/ENA/GenBank databases under the BioProject accession no. PRJNA730508 and the GenBank assembly accession no. GCA_019671135.1. The raw sequencing data were also deposited in the Sequence Read Archive database under accession no. SRX11233978.
